# Unraveling new functions of the Ca^2+^ sensor STIM1 in cell signaling

**DOI:** 10.1042/BST20250458

**Published:** 2026-05-06

**Authors:** Irene Sanchez-Lopez, Yolanda Orantos-Aguilera, Aida M. Lopez-Guerrero, Eulalia Pozo-Guisado, Francisco Javier Martin-Romero

**Affiliations:** 1 Department of Biochemistry and Molecular Biology, School of Life Sciences, Universidad de Extremadura, Badajoz, Spain; 2 Institute of Molecular Pathology Biomarkers, Universidad de Extremadura, Badajoz, Spain; 3 Department of Cell Biology, School of Medicine, Universidad de Extremadura, Badajoz, Spain

**Keywords:** calcium signalling, DNA damage response, endoplasmic reticulum, immune response, mitochondria, mitochondrial dysfunction

## Abstract

Calcium (Ca^2+^) signaling is a fundamental regulator of virtually all aspects of eukaryotic cell physiology, including gene expression, secretion, metabolism, motility, and cell fate decisions. The spatial and temporal control of cytosolic Ca^2+^ signals relies on a coordinated interplay between intracellular Ca^2+^ stores and plasma membrane (PM) Ca^2+^ channels. A critical advance in this field over the past two decades was the molecular identification of stromal interaction molecule 1 (STIM1) as the long-sought Ca^2+^ sensor that couples depletion of endoplasmic reticulum Ca^2+^ stores to Ca^2+^ influx across the PM. STIM1 has been established as a core component of store-operated Ca^2+^ entry, acting through direct activation of ORAI Ca^2+^ channels. However, accumulating evidence now indicates that STIM1 functions extend beyond this canonical role. STIM1 participates in the regulation of multiple classes of ion channels, contributes to the organization of membrane contact sites, and acts as a signaling scaffold influencing cellular processes independently of classical store depletion. This review summarizes the discovery and canonical functions of STIM1 and focuses on its emerging non-canonical roles, highlighting how STIM1 has evolved from an ER Ca^2+^ sensor into a multifunctional signaling hub.

## Discovery of STIM1 and identification as the ER Ca^2+^ sensor

The concept of store-operated calcium entry (SOCE), originally termed capacitative calcium entry, was formulated in 1986 to explain the observation that depletion of intracellular Ca^2+^ stores triggers sustained Ca^2+^ influx across the plasma membrane (PM) [1]. However, the molecular identity of the endoplasmic reticulum (ER) Ca^2+^ sensor remained elusive for more than a decade. In 2005, two independent RNA interference-based screens identified stromal interaction molecule 1 (STIM1) as an essential component of SOCE, acting as the missing link between ER Ca^2+^ content and PM Ca^2+^ entry. Roos and colleagues demonstrated that suppression of STIM1 protein expression, coded by the *STIM1* human gene (Ensembl ENSG00000167323), abolished Ca^2+^ release-activated Ca^2+^ (CRAC) currents [2], while Liou et al. showed that STIM1 rapidly relocalizes within the ER in response to Ca^2+^ store depletion [3]. STIM1 forms oligomers after Ca^2+^ store depletion and before the translocation of the protein to ER-PM junctions [4]. Using TIRF microscopy and patch-clamp, it was shown that STIM1 accumulates in discrete subregions of junctional ER located 10–25 nm from the PM, without detectable insertion of STIM1 into the PM [5], and this relocalization of STIM1 is determined by the status of Ca^2+^ concentration in the ER in close proximity to the PM [6].

Using cells from patients with severe combined immunodeficiency (SCID) that exhibited absent CRAC currents, Feske et al. then mapped the disease-causing mutation to ORAI1, and they showed that expression of wild-type ORAI1 restored CRAC/SOCE, thereby demonstrating that ORAI1 is the PM Ca^2+^ channel responsible for SOCE [7]. The physical interaction between the STIM1 C-terminal cytosolic region and ORAI1 C-terminus is required to trigger the opening of the Ca^2+^ channel [8]. The activation of SOCE allows the refilling of ER stores, and this refilling reverses both STIM1 multimerization and STIM1–ORAI1 interaction [9].

## Structural organization of STIM1 and mechanism of activation

**Figure 1:**
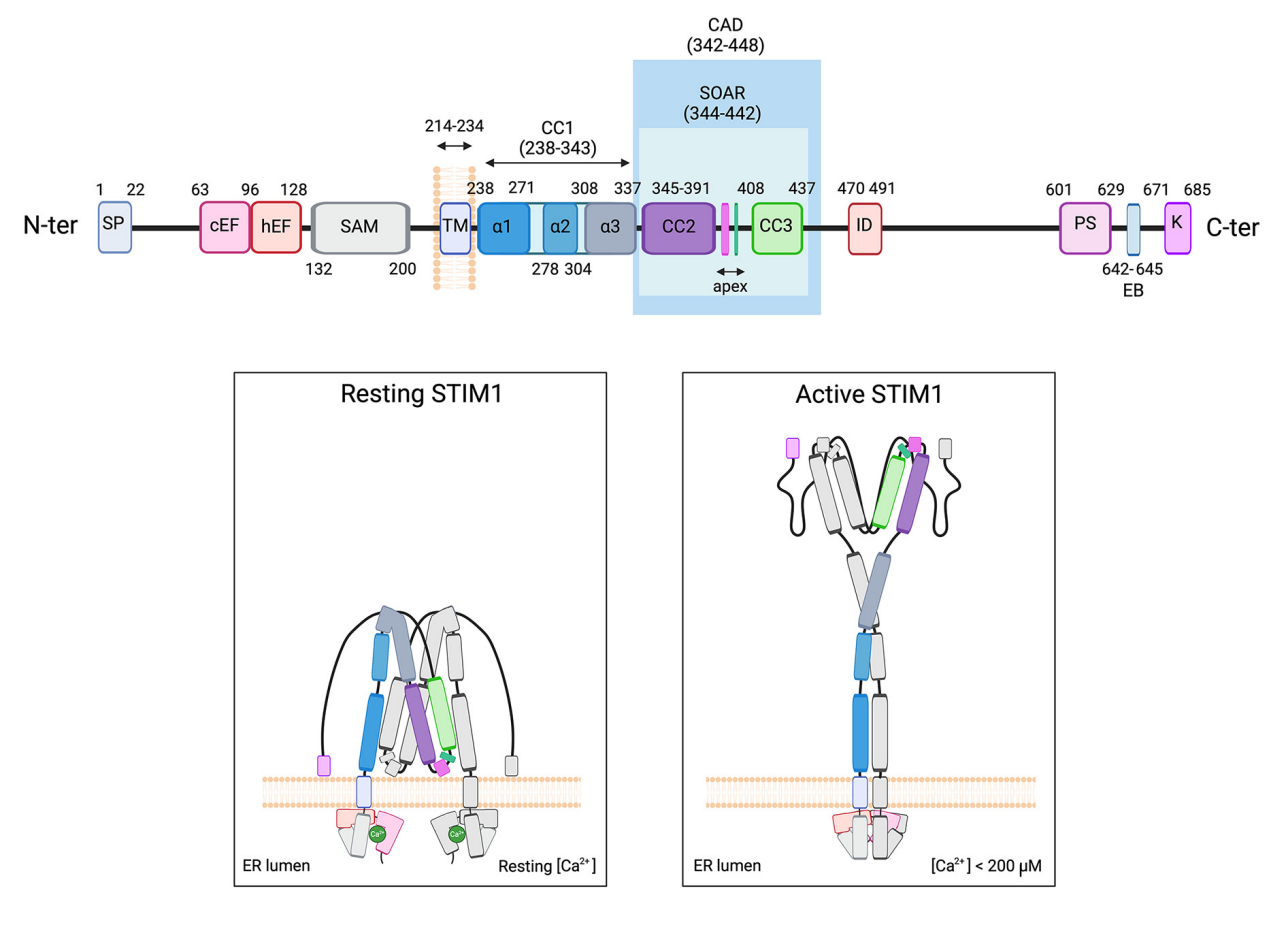
Domain organization and activation mechanism of STIM1 *Top panel:* Schematic representation of STIM1 domain architecture and its conformational rearrangement upon ER Ca^2+^ depletion. The N-terminal region located in the ER lumen contains the signal peptide, canonical EF-hand, hidden EF-hand, and SAM, which together function as the Ca^2+^-sensing module. A single transmembrane (TM) segment anchors STIM1 to the ER membrane. The cytosolic region comprises CC domains (CC1–CC3), including the STIM1–ORAI1 activating region (SOAR; residues 344–442), as well as an inhibitory domain, a proline/serine-rich region (PS), and a C-terminal polybasic (K) domain. *Bottom panel:* In resting conditions (left), Ca^2+^ binding to the EF-hand domains maintains STIM1 in a compact, inactive conformation through intramolecular interactions that mask the SOAR domain. Upon ER Ca^2+^ depletion (right), Ca^2+^ dissociation induces conformational changes that promote STIM1 oligomerization and extension, exposing the SOAR domain to enable interaction with ORAI1 channels at ER-PM junctions. This transition underlies the activation of store-operated Ca^2+^ entry (SOCE). Data adapted from [67,68]. Image created with BioRender.

STIM1 shows a modular domain architecture that underlies the transmission of the luminal Ca^2+^ levels information across the ER membrane towards its cytosolic domain (Figure 1). STIM1 N-terminal luminal region contains a canonical EF-hand Ca^2+^-binding motif paired with a non-canonical EF-hand and a sterile alpha motif (SAM) domain. Under resting conditions, Ca^2+^ binding stabilizes this luminal domain, maintaining STIM1 in an inactive conformation. Depletion of ER Ca^2+^ leads to Ca^2+^ dissociation from the EF-hand domain, triggering conformational rearrangements and oligomerization of STIM1 [10]. Thus, STIM1 is anchored in the ER membrane by a single transmembrane helix, which transduces luminal conformational changes to the cytosolic C-terminal region. This cytosolic portion (amino acids 235–685 for the canonical STIM1 isoform) contains several coiled-coil (CC) domains: CC1 (amino acids 238–343), CC2 (345–391), and CC3 (408–437) [11]. The CC1 contains 3 CC regions: CC1α1, amino acids 238–271; CC1α2, amino acids 278–304; and CC1α3, amino acids 308–337. In addition, the STIM1 cytoplasmic region shows a functional STIM1–ORAI activating region (SOAR; residues 344–442) or CRAC activation domain (CAD; residues 342–448) that interacts with and gates the ORAI channel [4,12–14]. In the resting state, the CC1α1 helix binds to the SOAR/CAD domain to form an autoinhibitory clamp that maintains STIM1 in a quiescent state [15]. The currently accepted model to explain inactive-to-active transition in STIM1 involves the luminal Ca^2+^ depletion in the ER, which is sensed through the EF-SAM domain. Dissociation of Ca^2+^ from the EF-SAM disrupts the intramolecular interaction between the EF-hands and SAM domains, causing aggregation of the luminal EF-SAM domains [16]. Luminal oligomer formation triggers structural changes that extend into the STIM1 transmembrane domain and are transmitted to the cytosolic region of STIM1, where they promote the release of SOAR from CC1. Consequently, the C-terminal domain undergoes a large-scale reorganization, transitioning into a more extended configuration that exposes both the SOAR/CAD domain (Figure 1). Active STIM1 proteins then assemble into higher-order multimers and translocate to ER-PM contact sites, where they physically bind and activate ORAI1 channels [16]. This interaction induces a conformational change in ORAI1 that opens the highly Ca^2+^-selective CRAC channel pore, allowing Ca^2+^ influx into the cell [17].

STIM1 has a closely related homolog, STIM2, which shares a broadly similar domain architecture and considerable functional overlap with STIM1, although several relevant differences have been reported. One notable distinction lies in the Ca^2+^-binding properties. STIM2 exhibits an approximately twofold lower Ca^2+^ affinity than that of STIM1 in the ER lumen [18]. Consequently, STIM2 can sense relatively small decreases in luminal Ca^2+^ concentration and can therefore respond to modest levels of store depletion, whereas STIM1 typically requires more substantial depletion before becoming activated. Both STIM1 and STIM2 bind ORAI1 through their C-terminal SOAR/CAD region. However, STIM2 acts as a comparatively weaker agonist. This difference has been attributed, at least in part, to a single amino acid substitution within the SOAR domain: Phe-394 in STIM1 is replaced by a leucine residue in the corresponding region of STIM2. This substitution is thought to underlie the reduced efficiency of ORAI1 activation by STIM2, resulting in slower and weaker store-operated Ca^2+^ entry compared with that mediated by STIM1 [19]. Given the space constraints of this mini-review, the discussion will primarily focus on STIM1.

## Physio(patho)logical roles of STIM1/ORAI1-mediated SOCE

SOCE mediated by STIM1 and ORAI1 is essential for numerous physiological processes. Although this is not a comprehensive review of the functions in which STIM1 is involved, the following are some examples. In T lymphocytes, SOCE sustains NFAT (nuclear factor of activated T-cells) activation for cytokine production, proliferation, and differentiation. B cells rely on SOCE for antibody class switching and plasma cell development; mast cells for degranulation and anaphylaxis; and neutrophils for phagocytosis and reactive oxygen species production [20]. Consequently, inherited loss-of-function (LoF) mutations in STIM1 or ORAI1 cause SCID, underscoring the role of this pathway in adaptive immunity [21]. Regarding muscle tissues, skeletal muscle uses SOCE to refill sarcoplasmic reticulum Ca^2+^ during repetitive contractions, regulate NFAT-mediated gene expression, and control cardiac hypertrophy [22,23]. The canonical SOCE function is so central that LoF mutations in STIM1 cause muscle weakness and reduced muscle mass [24], with prominent mitochondrial ultrastructural abnormalities in STIM1-KO muscles compared with ORAI1-KO mice, indicating a specific role of STIM1 in maintaining mitochondrial integrity in skeletal muscle [25]. In contrast, gain-of-function (GoF) mutations underlie conditions including tubular aggregate myopathy, or TAM [26]. When GoF mutations extend the phenotype beyond TAM, they produce Stormorken syndrome (TAM/STRMK), a disorder with skeletal muscle weakness and myalgia together with miosis, thrombocytopenia, hyposplenism, ichthyosis, short stature, and sometimes dyslexia [27–29].

STIM1, associated with its partner ORAI1, has a role in the proliferation of vascular smooth muscle cells, airway smooth muscle cells [30], endothelial progenitor cells [31], and numerous types of cancer cells (reviewed in [32]).

In neurons, STIM1–ORAI1 regulates dendritic spine morphology, long-term potentiation, and synaptic plasticity [33]. Interestingly, the PS2APP mouse model of Alzheimer’s disease (AD) shows a reduction of STIM1 at the onset of AD [34], similarly to what was reported for human postmortem medial frontal gyrus tissue from AD-diagnosed patients [35]. In this regard, long-term synaptic plasticity is recovered by STIM1 overexpression in astrocytes [34], pointing to a decrease in STIM1 expression levels as an early biomarker in AD.

While these examples illustrate the central physiological relevance of STIM1–ORAI1-mediated SOCE, a detailed discussion of the impact of LoF and GoF mutations in these proteins falls beyond the scope of this review. Readers interested in a comprehensive and up-to-date analysis of the physio(patho)logical roles of STIM1/ORAI1-mediated SOCE are referred to a recent dedicated review on this topic [36].

## Canonical interactions beyond ORAI1

One of the earliest indications that STIM1 functions extend beyond classical SOCE came from studies showing that STIM1 can regulate ion channels other than ORAIs. Members of the transient receptor potential canonical (TRPC) family, particularly TRPC1, TRPC4, and TRPC5, have been shown to interact with STIM1 [37]. In these contexts, STIM1 does not act as an ER Ca^2+^ sensor but functions as a gating or scaffolding protein that promotes channel activity. Unlike ORAI-mediated CRAC currents, TRPC channels generate non-selective cation currents, leading to different Ca^2+^ signaling signatures. STIM1 has also been reported to exert an inhibitory influence on certain voltage-gated Ca^2+^ channels, most notably the L-type Ca_V_1.2 channel [38,39]. Early studies demonstrated that following ER Ca^2+^ store depletion, STIM1 translocates to ER-PM junctions and directly interacts with Ca_V_1.2, suppressing channel activity. This inhibitory coupling provides a mechanism by which cells can prioritize SOCE while limiting voltage-dependent Ca^2+^ influx. Such regulation could be particularly relevant in excitable cells, including neurons and vascular smooth muscle cells, where modulation of Ca_V_ channels influences excitability, contractility, and gene expression. Nevertheless, the physiological significance and the mechanisms underlying this regulation remain debated, as the functional interplay between STIM1 and voltage-operated Ca^2+^ channels appears to be highly context- and cell-type-dependent. For example, in mouse hippocampal neurons, knockdown of STIM1 or STIM2 did not increase depolarization-induced Ca^2+^ influx, and in HEK cells, neither Ca^2+^ store depletion nor STIM1 overexpression inhibited Ca_V_1.2 currents [40]. These observations suggest that STIM1-mediated inhibition of L-type channels may not be a universal mechanism and could depend on specific cellular environments or regulatory partners.

Although STIM1 is classically activated by ER Ca^2+^ depletion, several lines of evidence suggest that STIM1 can exert functional effects independently of ER Ca^2+^ depletion. STIM1 exhibits store-independent activity in regulating arachidonate-regulated Ca^2+^-selective (ARC) channels, where it controls Ca^2+^ entry through these channels without ER store depletion [41]. STIM1 also mediates store-independent activation of ORAI1 by SPCA2 in secretory epithelial cells to set basal cystic fibrosis transmembrane conductance regulator Cl^−^ channel, bypassing traditional ER Ca^2+^ depletion [42].

Beyond its role in ion channel regulation, STIM1 contributes to the physical organization of intracellular membranes. STIM1 interacts with microtubule plus-end-binding proteins, such as EB1 [43] (a.k.a. MAPRE1), linking ER dynamics to the cytoskeleton. This interaction allows STIM1 to influence ER morphology and positioning. Asanov and colleagues proposed a model in which the depletion of the ER triggers the dissociation of the STIM1-EB1 complex in the growing microtubule [44], a dissociation that is enhanced by the ERK1/2-dependent phosphorylation of STIM1 in the vicinity of its EB1-binding domain, in the C-terminus [45]. Subsequently, STIM1 associates with adenomatous polyposis coli protein, and this complex stabilizes STIM1 puncta near the ER-PM junctions, allowing the activation of ORAI channels [44]. Ca^2+^ entry triggers STIM1 dephosphorylation and binding back to EB1 [45], a binding that is considered a trapping mechanism to restrict STIM1 translocation to ER-PM junctions [46].

STIM1's canonical role in SOCE and calcium signaling has dominated research for years. However, a growing body of evidence reveals non-canonical functions that extend beyond classical calcium entry. These emerging roles reflect the multifunctional nature of STIM1 and its potential integration into other cellular processes.

## Involvement in STING-cGAS pathway as a novel non-canonical function

**Figure 2:**
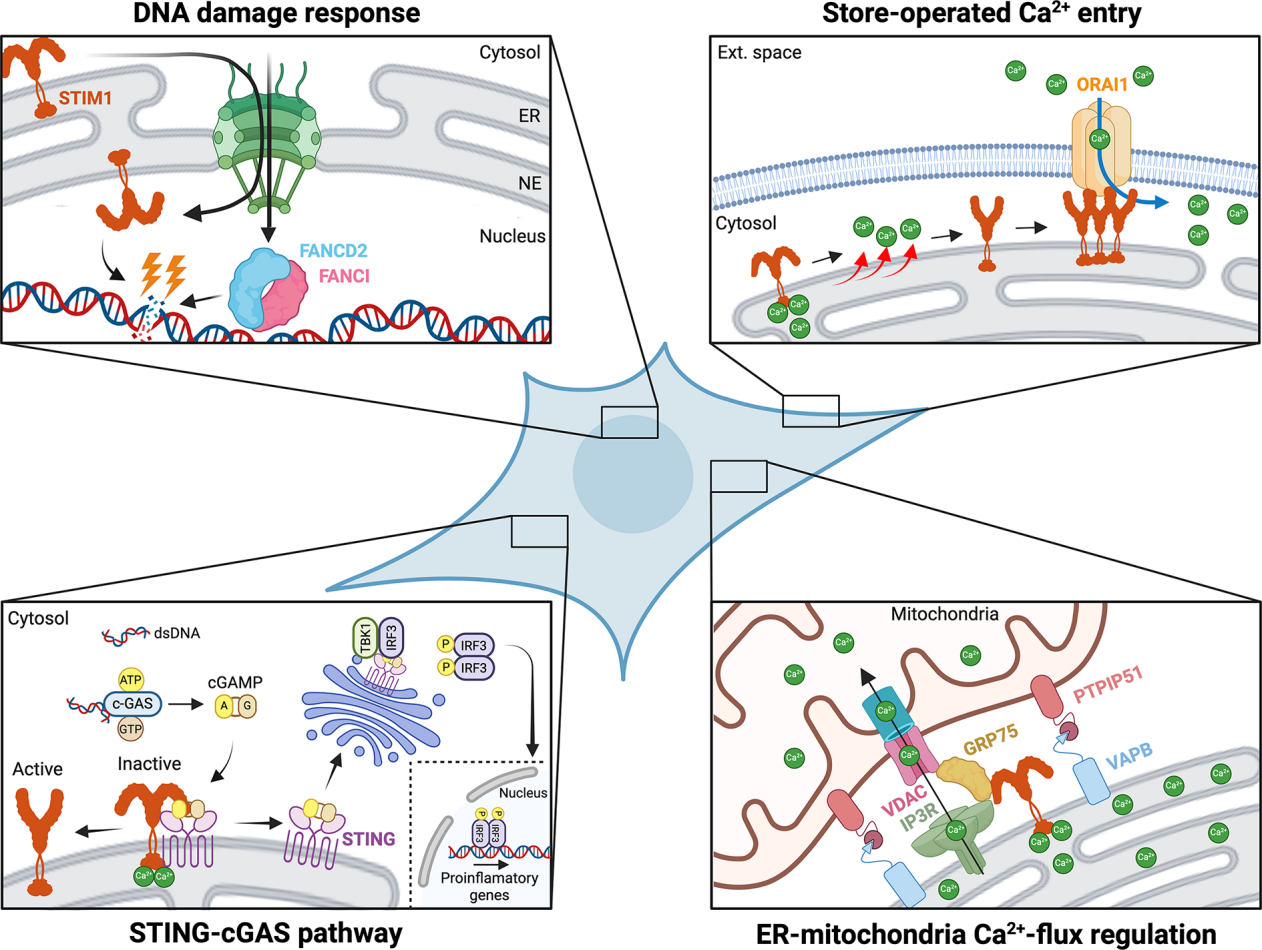
Canonical and non-canonical functions of STIM1 This model illustrates STIM1 as a central signaling hub coordinating canonical SOCE with non-canonical roles in DNA repair, immune signaling, and ER-mitochondria coupling. (*Top right*) In its canonical function, ER Ca^2+^ depletion promotes STIM1 activation and coupling to the PM Ca^2+^ channel ORAI1, resulting in SOCE and elevation of cytosolic Ca^2+^ levels. (*Top left*) STIM1 is shown at nuclear envelope (NE), where it is associated with DNA damage responses (DDR). DNA double-strand breaks are illustrated together with recruitment of FANCD2 and FANCI, highlighting a proposed role for STIM1 in genome stability maintenance. (*Bottom left*) STIM1 intersects with innate immune signaling pathways. Under resting conditions, STING is retained in the ER via its interaction with STIM1, thereby preventing its activation. However, cytosolic DNA sensing by cGAS generates cGAMP from ATP and GTP, leading to activation of STING at the ER membrane and downstream immune response signaling. (*Bottom right*) At ER-mitochondria contact sites, STIM1 contributes to inter-organelle communication. The IP_3_R–VDAC complex, bridged by GRP75, facilitates Ca^2+^ transport from the ER to mitochondria. Image created with BioRender.

The identification of STIM1 interaction partners outside the classical SOCE machinery has provided initial clues supporting STIM1’s functional diversification. In 2019, Srikanth et al. showed that STIM1 has an additional role as a negative regulator of the innate immune response by modulating the cGAS-STING pathway [47]. In this pathway, the DNA sensor cyclic GMP–AMP synthase (cGAS) detects DNA released into the cytosol and catalyzes the production of the second messenger 2′,3′-GAMP (cyclic GAMP or cGAMP). This cyclic dinucleotide activates the signaling adaptor protein stimulator of interferon genes (STING) at the ER. Upon activation, STING translocates from the ER to the ER-Golgi intermediate compartment, where it recruits TANK-binding kinase 1 (TBK1) and interferon regulatory factor 3 (IRF3). TBK1-mediated phosphorylation of IRF3 promotes IRF3 dimerization and nuclear translocation, ultimately inducing the transcription of type I interferons (IFNs) [48,49] (see Figure 2).

In this context, STIM1 has been reported to function as an “ER retention factor” that limits basal activation of the pathway. Through direct interaction, primarily mediated by their N-terminal and transmembrane domains, STIM1 retains STING at the ER membrane, thereby preventing its spontaneous activation [47]. Consistent with this model, loss of STIM1 in cells from a human SCID patient results in constitutive STING activation at rest [46].

Although this regulatory function appears to be independent of classical SOCE, as ORAI1 deficiency does not enhance type I IFN responses [46], the Ca^2+^-sensing properties of STIM1 may still contribute to this process. Indeed, the conformational changes that STIM1 undergoes following ER Ca^2+^ store depletion reduce its ability to retain STING at the ER [47,50]. However, very recent findings indicate that the precise contribution of STIM1 to this regulatory mechanism should be interpreted with caution. In particular, STIMATE, a well-established interactor of STIM1 [51], has been identified as an ER-resident suppressor of STING that can regulate STING signaling independently of STIM1 [52]. These observations raise the possibility that the previously proposed retention function attributed to STIM1 may, at least in part, reflect the activity of associated regulatory proteins, highlighting the need for further studies to clarify the exact molecular contribution of STIM1 in controlling STING activation.

## STIM1 interactome reveals novel functions

The growing body of information on the STIM1 interactome has enabled the study of additional, potentially non-canonical functions of STIM1. Analysis of these interactions using public databases such as BioGRID reveals that some high-throughput datasets report interactions with mitochondrial and nuclear proteins. These findings suggest that STIM1 may be part of ER molecular complexes that interact with mitochondria and may also have a nuclear role.

### STIM1 regulates Ca^2+^ transfer between ER and mitochondria

A more detailed analysis of the proteome at ER-mitochondria contact sites reveals the presence of STIM1 in these interfaces, also known as mitochondria-associated ER membranes (MAMs) [53]. This localization of STIM1 has been further supported recently through dedicated localization experiments using STED microscopy [54], MAM isolation and low-throughput approaches, including immunoblotting, proximity ligation assays, and reconstitution of dimerization-dependent fluorescent protein [55].

The finding that STIM1, a Ca^2+^ sensor, is localized at MAMs prompted us to investigate its potential function at this unique subcellular site. Regulation of mitochondrial Ca^2+^ concentration is critical for mitochondrial function, as Ca^2+^ acts as a positive regulator of the Krebs cycle oxidoreductases [56]. Accordingly, an adequate flux of Ca^2+^ into the mitochondrial matrix is required to sustain the generation of reducing equivalents and support mitochondrial electron transport. Under resting conditions, mitochondrial free Ca^2+^ concentration is similar to cytosolic levels (∼100–200 nM), but it can increase by 10- to 20-fold upon stimulation [57]. The primary pathway for Ca^2+^ entry into the mitochondrial matrix is the mitochondrial calcium uniporter complex (mtCU). However, due to its low Ca^2+^ affinity, efficient Ca^2+^ uptake through the mtCU requires the formation of high-Ca^2+^ microdomains. These microdomains are generated at the ER surface, and MAMs facilitate efficient Ca^2+^ transfer from the ER to mitochondria. This transfer is mediated by a molecular complex composed of the inositol 1,4,5-trisphosphate receptor (IP_3_R) at the ER, the voltage-dependent anion-selective channel (VDAC) at the outer mitochondrial membrane, and GRP75 (also known as HSPA9), which physically and functionally links the ER and mitochondria [58,59].

A recent study from our group reported that STIM1 acts as a Ca^2+^ sensor at ER-mitochondria contact sites, where it regulates the function of the IP_3_R–GRP75–VDAC complex by interacting with GRP75 in a conformation-dependent manner. Under resting conditions, when STIM1 adopts a folded conformation associated with high intraluminal ER Ca^2+^ levels, STIM1 binds to GRP75, thereby maintaining ER-mitochondria tethering and enabling efficient Ca^2+^ transfer between the two organelles. However, upon depletion of ER luminal Ca^2+^, resulting from Ca^2+^ release toward ER-mitochondria contact sites, STIM1 undergoes a conformational transition to a more extended structure (Figure 1), as described above. This conformational change leads to dissociation of the STIM1–GRP75 interaction, reduced levels of both proteins in MAMs, and termination of Ca^2+^ transfer (see Figure 2). Importantly, this mechanism prevents mitochondrial Ca^2+^ overload [55], highlighting STIM1 as an efficient regulator of Ca^2+^ flux at ER-mitochondria interfaces. Consistently, STIM1-deficient (STIM1-KO) cells exhibit impaired Ca^2+^ transfer to mitochondria. Significantly, cells expressing STIM1 variants lacking the GRP75-interaction domain (amino acids 551–611) also display defective mitochondrial Ca^2+^ transfer, even though SOCE remains intact, indicating that SOCE alone is insufficient to support mitochondrial Ca^2+^ uptake. This defect results in lower basal mitochondrial Ca^2+^ levels, compromised maximal mitochondrial respiration, and reduced ATP production [55].

The most challenging aspect of the present study lies in the identification of the interaction domain, as this required the use of deletion mutants—namely, STIM1 variants harboring different deletions in its C-terminal sequence—to define the minimal region necessary for this interaction. This approach is currently considered appropriate because the region spanning amino acids 485–685 of STIM1 is highly flexible and contains several intrinsically disordered regions, whose structural plasticity enables dynamic, context-dependent protein–protein interactions while rendering their structural characterization inherently difficult [60].

Changes in STIM1 levels at MAMs following store depletion have also been reported by García Casas et al. [54]. In that study, an increase in STIM1 at ER-mitochondria contact sites was observed upon Ca^2+^ store depletion, and this enrichment appeared to require the participation of STIMATE, a known interactor of STIM1 that regulates its activation and SOCE. This increase preceded the rise in ER-mitochondria juxtaposition, a model that is consistent with, and does not contradict, the role of STIM1 as an ER-to-mitochondria Ca^2+^ flux regulator proposed by our group [55]. However, the study by García Casas et al. [54] mainly reported the presence and redistribution of STIM1 at these contact sites, while the molecular mechanisms underlying this observation were not addressed.

### STIM1 has a role in DNA damage response

On the other hand, initial three-dimensional reconstructions of microscopy images revealed the presence of STIM1, SERCA2a, and ORAI1 at the NE of cardiomyocytes, as well as within invaginations of this structure [61]. However, the resolution of the technique used did not allow discrimination between the inner and outer nuclear membranes. Nevertheless, the possibility that STIM1 could have a specific role at the NE was later suggested by the analysis of the STIM1 interactome. This analysis revealed interactions with several nuclear proteins as well, including multiple importins (IPO4, IPO7, IPO8, IPO9, and IPO11), nuclear export proteins such as XPO1, XPO5, and XPOT, the nuclear pore complex protein NUP205, and the inner nuclear membrane protein emerin [62]. Although the occurrence of STIM1 at the NE was confirmed by independent groups [62,63], we reported that STIM1 is present at the inner nuclear membrane and that its nuclear levels increase following DNA damage caused by interstrand cross-links (ICLs) [62]. This nuclear accumulation is mediated by a canonical nuclear localization signal (NLS; residues 382-KIKKKR-387) located in the CC2 domain. As described above, CC2 and CC3 together form an approximately 110-amino acid region required for ORAI1 binding during SOCE and adopt a hairpin structure that is folded over the CC1 domain in the resting state. Upon Ca^2+^ store depletion, this structure becomes extended, and the conformational change allows the NLS to become accessible to other interacting proteins. Notably, this NLS does not play a critical role in maintaining the closed conformation, as mutation of residues 382-KIKKK-386 to 382-QIQQQ-386 does not cause extension of the cytosolic domain [64]. This finding suggests that the NLS does not compete with other functions previously described for the CC1-CC2-CC3 domains of STIM1, consistent with the ability of the 382-AIAAAA-387 mutant to rescue SOCE when overexpressed in STIM1-KO HEK293 cells [62].

Nuclear STIM1 plays a pivotal role in DNA damage repair, particularly in the response to ICLs and replication stress, as evidenced by its co-precipitation with the FANCD2–FANCI heterodimer. STIM1 deficiency reduces nuclear FANCD2 levels and chromatin-associated foci, thereby impairing the Fanconi anemia pathway response to MMC-induced ICLs [62]. Given its nature as an ER protein, it is plausible that STIM1 facilitates the relocalization of DNA damage foci to the nuclear periphery, although the underlying mechanism is not known, representing a hypothesis that remains to be tested. Consistent with this, STIM1-deficient cells exhibit increased sensitivity to replication inhibitors (hydroxyurea and aphidicolin), elevated number of 53BP1 nuclear bodies, and defective FANCD2/BRCA1 foci, indicating homologous recombination (HR) repair deficiencies in S-phase [62].

Consistent with a nuclear role and localization, *STIM1^+/D84G^* mice, carrying a GoF mutation associated with a progressive decline in locomotor activity and muscle loss, display alterations in nuclear morphology, NE integrity, and increased DNA damage. These observations led to the proposal that constitutively active STIM1 mutations may contribute to the pathogenesis of STIM1-associated myopathies [63], although the exact mechanism underlying this relationship remains unclear.

Recent work has revealed an unexpected link between intracellular Ca^2+^ signaling, cytosolic DNA sensing, and the maintenance of genome stability during replication stress. Replication stress can generate fragments of cytosolic DNA that are detected by the innate immune sensor cGAS, leading to activation of the cGAS-cGAMP-STING signaling pathway, described above. In this context, STING signaling promotes Ca^2+^ release from the ER through TRPV2 channels, increasing cytosolic Ca^2+^ levels [65]. The resulting Ca^2+^ signal activates the CaMKK2-AMPK kinase cascade, which suppresses the nuclease EXO1 and thereby prevents excessive processing of stalled replication forks. Through this mechanism, cytosolic DNA sensing is coupled to Ca^2+^-dependent signaling pathways that protect replication forks and maintain genome integrity. Importantly, this pathway operates in parallel to the canonical ATR/Chk1 replication stress checkpoint and represents an additional layer of genome surveillance linking DDR, innate immune signaling, and intracellular Ca^2+^ dynamics [66].

However, despite these advances, a unified mechanistic framework that reconciles findings supporting a role for nuclear STIM1 [62] with the STING-dependent Ca^2+^ signaling pathway described in [65] has yet to be established.

## Perspectives

- STIM1 is a central regulator of calcium signaling, a process fundamental to cellular physiology and organismal homeostasis. Beyond its canonical role in SOCE, STIM1 is increasingly recognized as a multifunctional protein whose dysregulation contributes to immunodeficiency, myopathy, neurodegeneration, inflammation, and cancer, underscoring its broad biological and clinical relevance.
- Current evidence supports a model in which STIM1 functions as a dynamic signaling hub whose conformational plasticity enables context-dependent interactions. In addition to activating ORAI channels, STIM1 regulates other ion channels, organizes membrane contact sites, modulates innate immune signaling, controls ER-mitochondria Ca^2+^ transfer, and participates in nuclear DDR, integrating Ca^2+^ sensing with diverse cellular pathways (see Figure 2).
- Key challenges include defining how STIM1 selectively engages distinct partners across cellular compartments and determining the *in vivo* relevance of its non-canonical functions. How are the different pools of STIM1 spatially and temporally regulated to avoid interference between canonical and non-canonical functions? To what extent do post-translational modifications, oligomerization states, or alternative interaction partners bias STIM1 toward specific signaling outputs? Moreover, the involvement of STIM1 in mitochondrial bioenergetics, DNA damage repair, and immune signaling pathways suggests that dysregulation of its non-canonical roles may contribute to human disease beyond classical Ca^2+^ signaling disorders. Addressing these questions will require integrated approaches combining structural biology, high-resolution imaging, and systems-level interactomics. Ultimately, a deeper understanding of STIM1 as a multifunctional signaling hub may enable targeted modulation of specific STIM1 activities for therapeutic benefit without disrupting essential SOCE.

## Open Access

Open access for this article is provided via our Subscribe to Open programme, made possible through the support of our subscribing institutions. Learn more here: https://portlandpress.com/pages/open_access_options_and_prices#conditional.

## Abbreviations

AD: Alzheimer’s disease
CRAC: Ca^2+^ release-activated Ca^2+^
cGAS: cyclic GMP–AMP synthase
CC: coiled-coil
DDR: DNA damage response
ER: endoplasmic reticulum
GoF: gain-of-function
IRF3: interferon regulatory factor 3
IFNs: interferons
ICLs: interstrand cross-links
LoF: loss-of-function
MAMs: mitochondria-associated ER membranes
mtCU: mitochondrial calcium uniporter complex
NE: nuclear envelope
NFAT: nuclear factor of activated T-cells
PM: plasma membrane
SCID: severe combined immunodeficiency
SOCE: store-operated Ca^2+^ entry, store-operated calcium entry
STIM1: stromal interaction molecule 1
SAM: sterile alpha motif
TBK1: TANK-binding kinase 1
TRPC: transient receptor potential canonical
VDAC: voltage-dependent anion-selective channel
